# Current or recent malaria infection is associated with elevated inflammation-adjusted ferritin concentrations in pre-school children: a secondary analysis of the BRINDA database

**DOI:** 10.1017/S0007114524002319

**Published:** 2024-10-28

**Authors:** Fanny Sandalinas, Amy MacDougall, Suzanne Filteau, Heidi Hopkins, Tineka Blake, Hanqi Luo, Parminder S. Suchdev, Laird Ruth, Melissa F. Young, Edward J. M. Joy

**Affiliations:** 1 Faculty of Epidemiology and Population Health, London School of Hygiene & Tropical Medicine, Keppel Street, London WC1E 7HT, UK; 2 Faculty of Infectious and Tropical Diseases, London School of Hygiene & Tropical Medicine, Keppel Street, London WC1E 7HT, UK; 3 School of Biosciences, University of Nottingham, Nottingham NG7 2RD, UK; 4 Hubert Department of Global Health, Emory University, 1518 Clifton Road NE, Atlanta, GA, USA; 5 Centers for Disease Control and Prevention, 4770 Buford Highway NE, Atlanta, GA, USA

**Keywords:** Malaria infection, Inflammation, Ferritin

## Abstract

Inflammation and infections such as malaria affect micronutrient biomarker concentrations and hence estimates of nutritional status. It is unknown whether correction for C-reactive protein (CRP) and *α*1-acid glycoprotein (AGP) fully captures the modification in ferritin concentrations during a malaria infection, or whether environmental and sociodemographic factors modify this association. Cross-sectional data from eight surveys in children aged 6–59 months (Cameroon, Cote d’Ivoire, Kenya, Liberia, Malawi, Nigeria and Zambia; *n* 6653) from the Biomarkers Reflecting Inflammation and Nutritional Determinants of Anaemia (BRINDA) project were pooled. Ferritin was adjusted using the BRINDA adjustment method, with values < 12 μg/l indicating iron deficiency. The association between current or recent malaria infection, detected by microscopy or rapid test kit, and inflammation-adjusted ferritin was estimated using pooled multivariable linear regression. Age, sex, malaria endemicity profile (defined by the *Plasmodium falciparum* infection prevalence) and malaria diagnostic methods were examined as effect modifiers. Unweighted pooled malaria prevalence was 26·0 % (95 % CI 25·0, 27·1) and unweighted pooled iron deficiency was 41·9 % (95 % CI 40·7, 43·1). Current or recent malaria infection was associated with a 44 % (95 % CI 39·0, 52·0; *P* < 0·001) increase in inflammation-adjusted ferritin after adjusting for age and study identifier. In children, ferritin increased less with malaria infection as age and malaria endemicity increased. Adjustment for malaria increased the prevalence of iron deficiency, but the effect was small. Additional information would help elucidate the underlying mechanisms of the role of endemicity and age in the association between malaria and ferritin.

Micronutrient deficiencies underlie a large disease burden, especially in low-income countries^(1)^. Iron deficiency is estimated to affect 1·5–2 billion people worldwide^(2)^, with considerable adverse health effects as iron is needed for energy production, oxygen transport and utilisation, cellular proliferation and pathogen destruction^(3)^. Iron deficiency affects all populations, but the most vulnerable are women and children due to their greater requirements^(4)^.

Various biomarkers can be used to assess iron status. Serum ferritin is a measure of the amount of iron in body stores and is a positive acute phase protein. During the inflammatory process, its concentration increases independently of iron stores. This is associated with body iron redistribution. This process, mediated by hepcidin, results from the storage of serum iron, the reduction of iron absorption from the diet and a reduced release of iron from body stores^(5)^. This can confound the proper assessment of iron deficiency in individuals experiencing inflammation. A correction method was developed by the Biomarkers Reflecting Inflammation and Nutritional Determinants of Anaemia (BRINDA) group, whereby a linear regression is used to adjust the biomarker concentration by the concentrations of C-reactive protein (CRP) and *α*1-acid glycoprotein (AGP) in serum^(6)^. In 2020, the WHO updated the guideline on using ferritin concentrations to assess iron status in people and populations – and included malaria as a possible independent factor for adjustment^(7)^.

Malaria is a leading cause of morbidity and mortality in children. According to the WHO, there were 247 million cases of malaria in 2021, and 95 % of these occurred in Africa^(8)^. *Plasmodium falciparum* accounted for 99·7 % of infections in sub-Saharan Africa^(9)^. The level of malaria endemicity is defined as the degree of malaria transmission in an area^(10)^, and the parasite prevalence in children aged 2–10 years is commonly used to define endemicity levels^(11)^. Naturally acquired antibody responses against *P. falciparum* require repeated parasite exposure to attain protection. The rate of antibody acquisition against *P. falciparum* proteins is influenced by various factors, including age of the human host, transmission intensity and the antigen type. In general, antibody levels increase with both age and higher transmission intensity (usually defined by the parasite prevalence in young children)^(12)^.

Currently used malaria diagnostic methods include microscopy, rapid diagnostic tests (RDT) and Polymerase Chain Reaction (PCR). There are three antigens commonly targeted by RDT, including histidine-rich protein 2 (HRP2). HRP2 is specific to *P. falciparum.* HRP2-based assays can detect persistent antigenemia for up to several weeks after parasites have been eradicated, while other RDT and microscopy detect only current infections^(13,14)^. There are implications for the interpretation of ferritin values, as ferritin concentrations can stay elevated in the blood for several weeks after parasite clearance^(15)^.

A recent study in Burkina Faso showed that asymptomatic malarial infections in young children in high-prevalence areas, measured by the presence of serum HRP2 and the absence of fever, had an additive effect to elevated AGP and CRP on serum ferritin; this resulted in inaccurate estimates of iron deficiency prevalence when only AGP and CRP were considered^(16)^. It is likely that ferritin concentration can be increased during malaria infection independently of inflammation, although the specific pathways are not yet fully understood^(17)^.

In 2017, Namaste *et al*. conducted a multi-country analysis of the effect of malaria on the interpretation of ferritin using the BRINDA datasets in pre-school children (PSC) and women of reproductive age^(5)^. BRINDA datasets comprise nationally or regionally representative surveys on micronutrient biomarkers status, primarily conducted in low- and low-middle-income countries. Namaste *et al*. found that malaria infection was independently associated with ferritin after adjusting for CRP and AGP. However, the researchers concluded that when measuring CRP and AGP, there appears to be limited utility in measuring malaria status to adjust ferritin concentrations – because malaria adjustments alone, or in addition to CRP and AGP adjustments, did not considerably change the estimates of iron deficiency prevalence. A subsequent analysis by Luo *et al*. also suggested that malaria should not be included in the BRINDA inflammation adjustment method as a binary variable^(18)^. In these two analyses led by Namaste and Luo, malaria species, diagnostic methods, endemicity profile or stage of infection were not studied, although these factors could modify the association between malaria and ferritin concentrations^(19)^.

This study’s objective was to verify our assumption that the current adjustment for ferritin using CRP and AGP does not fully capture the modification in ferritin concentrations during a malaria infection and to test whether environmental and sociodemographic factors – including acquired immunity, reflected by malaria endemicity and age – can modify this association. The findings may support the development of a new method to adjust ferritin in the context of malaria and ultimately result in more accurate estimates of iron deficiency prevalence.

## Methods

### Biomarker datasets

Micronutrient biomarker data from national and regional surveys were accessed from the BRINDA project (www.BRINDA-nutrition.org). The protocol was reviewed and approved by the London School of Hygiene & Tropical Medicine (LSHTM) ethics review committee. The methods for identifying datasets, inclusion and exclusion criteria and data management for the BRINDA project have been described in detail elsewhere^(20)^. The surveys were nationally or regionally representative, and the BRINDA inclusion criteria were surveys that (1) were conducted after 2004, (2) had target groups including PSC, women of reproductive age or both, and (3) used a similar laboratory methodology for the measurement of at least one biomarker of iron or vitamin A status and at least one biomarker of inflammation (AGP or CRP). We included only surveys of PSC for which the following were available: (1) the measurement of ferritin and at least one inflammation marker (AGP or CRP) and (2) a measure of malaria infection by a standardised method.

Among the thirty datasets available for PSC, ten had information on current or recent malaria infection. However, one of them (Burkina Faso) used retrospective measurement of malaria antibodies in serum to define infection^(21)^. This method is unlikely to detect only recent or current infections; thus, the dataset was not included in our database. The 2016 survey from Nepal did not detect any malaria case and was not included. In total, 7886 PSC from eight surveys were available for inclusion (online Supplementary Table 1). Nationally representative survey data were included from Cote d’Ivoire in 2007^(22)^, Cameroon in 2009^(23,24)^, Liberia in 2011^(25)^ and Malawi in 2016^(26)^. Regional survey data were included from Kenya in 2007^(27)^ and 2010^(28)^, Nigeria in 2012^(29)^ and Zambia in 2009^(30)^. Only diagnosed infections with *P. falciparum* were included in the analysis.

Ferritin, AGP and CRP concentrations were assessed with the use of a sandwich ELISA at the VitMin Laboratory^(31)^ in all surveys – apart from Zambia, where the same analytical method was used in the Tropical Diseases Research Centre in Ndola. Current malaria infection was assessed with microscopy in Kenya (both surveys), Cote d’Ivoire, Nigeria and Zambia. Current or recent malaria infection was assessed with the Paracheck Pf rapid-diagnostic test (Orchid Biomedical System) in Liberia, the Malaria Ag CELISA kit (Cellabs Pty, Ltd) in Cameroon and the BIOLINE Malaria Ag *P.f*/Pan in Malawi. These three kits detect the presence of HRP2, a protein specific to *P. falciparum* ^(9)^.

### Inflammation adjustment

For each dataset individually, ferritin values were adjusted for inflammation using the regression approach with the BRINDA package^(32)^. The regression approach has been described in detail elsewhere^(5)^ and uses linear regression to adjust ferritin concentrations by the CRP and AGP concentrations on a continuous scale. All the ferritin observations that have a corresponding CRP value, and/or AGP value above the first decile of the considered biomarker, were adjusted with linear regression. We decided to use the external deciles^(20)^ instead of individual survey deciles for consistency and because two of the individual surveys (Cote d’Ivoire and Zambia) had a very low first decile of CRP, suggesting a low level of inflammation.

### Endemicity profile

When assessing malaria endemicity, parasite rate data constitute most of the global information available^(33)^ and are preferable to using prevalence data from surveys, which are likely to vary according to multiple factors including the survey timing and the seasonality of transmission. A malaria endemicity profile was therefore assigned to each study, considering the year the study was conducted and the study location (Table 1), using infection prevalence data extracted from the database hosted by the Malaria Atlas Project^(34)^. It was not possible to identify subnational levels in our dataset, as the region or district names had been removed from the database. Therefore, subnational variations in endemicity were not considered. The data in the Malaria Atlas database were reported from 2010; therefore, the value for the year 2010 was used for surveys conducted before 2010. This was the case for Cameroon, Kenya, Zambia and Cote d’Ivoire. For these countries, other data sources on malaria prevalence were consulted, such as Demographic and Health Survey reports, to confirm the prevalence data; no discrepancies were noted^(35)^. The different categories were defined by the WHO^(11)^: very low intensity (PfP < 1 %), low intensity (PfP ≥ 1 % and < 10 %), moderate intensity (PfP ≥ 10 % and < 35 %) and high intensity (PfP ≥ 35 %).

**Table 1.** Endemicity profile of the survey settings from eight datasets from the BRINDA database in malaria endemic countries in Africa

Study (location, year)	Reported endemicity (location, year)
**Moderate endemicity** PfP ≥ 10 % and < 35 %	
Cameroon (national, 2009)	Most regions: > 10 % and < 35 %, 2009
Kenya (Nyando Division, Nyanza Province, 2007)	15 % (Kisumu, 2010)
Kenya (Nyando Division, Nyanza Province, 2010)	15 % (Kisumu, 2010)
Nigeria (Akwa Ibom State, 2012)	30 % (Akwa Ibom, 2012)
Malawi (National, 2016)	All regions: > 10 % and < 35 %, 2016
Zambia (Central and Eastern provinces, 2009)	22 % (Eastern,2010) 8 % (Central, 2010)*
**High endemicity** PfP ≥ 35 %	
Cote d’Ivoire (National, 2007)	Most regions: > 35 %, 2010
Liberia (National, 2011)	Most regions: > 35 %, 2011

PfP, *Plasmodium falciparum* infection prevalence.

Data were extracted from the Malaria Atlas Project database^(34)^.

* Zambia was considered moderate as one province was in the moderate category and the other province was in the upper end of the low endemicity category.

In three of these datasets (Cote d’Ivoire, Kenya 2007 and Kenya 2010), additional information on the number of *P. falciparum* parasites was available. This was then converted into a parasitaemia level, which is the number of parasites per microlitre of blood, on the basis of 8000 leucocytes in one microlitre of blood. A low level of parasitaemia was defined as < 1000 parasites per microlitre of blood, based on previous publications^(36)^.

### Statistical analysis

The outcome variable, inflammation-adjusted serum ferritin, was continuous. The exposure variable, malaria infection, was a binary variable (infected, uninfected). We examined the data to check for missing data, errors and inconsistencies and to gain an understanding of the distributions and patterns among the variables. We included only children who had a result for a malaria test and ferritin values. In the data, we examined the associations between the exposure and each potential confounder and potential effect modifier (age, sex, rurality, malaria endemicity profile and malaria diagnostic method), and between the outcome and each of these potential confounders and effect modifiers. Complex survey weights were accounted for when analysing individual data. Weights were not applied for pooled estimates, as the pooled dataset is used to assess a biological association and is not meant to be representative at any regional level; therefore, weights were also not used for the linear models which draw on the pooled dataset.

### Linear model

A pooled multivariable linear regression analysis was conducted to estimate the association between malaria and inflammation-adjusted ferritin. To account for the clustering effect by country in the pooled database, we included the survey identifier in the model as a fixed effect. The dependent variable serum ferritin was transformed (natural log) to improve the original skewed distribution. Consequently, the regression model was built on the logarithmic scale. If the estimated coefficient for malaria is *β̂*, then a malaria infection was associated with a 100 × (*e* ^*β̂*^ − 1) = x percent change in ferritin. The crude association between malaria and inflammation-adjusted ferritin was assessed. The model was then adjusted for each potential confounder, as follows: (1) individual characteristics such as age (categorical, in age group: < 2 years and > 2 years), sex, rural or urban location, and (2) variables likely to modify the relation between ferritin and malaria (malaria endemicity profile, malaria diagnostic method, age, sex and residence). A new model was created after adding each of these confounders and effect modifiers, and all the results of the different models are presented in online Supplementary Table 2. The confounders were selected based on plausible biological mechanisms and previously observed associations. Decisions about whether to include potential confounders in the final model were made according to whether their inclusion changed the effect estimate for the main exposure. Therefore, model A was chosen as the model for the analysis of the main effect of malaria on inflammation-adjusted ferritin, while model M was chosen as the final model with interactions. We limited the list of potential cofounders or effect modifiers to those available for all countries. For example, the presence of fever in the last 24 h, an important variable to define the stage of malaria infection, was available in only three datasets. Maternal education was not available for two of the datasets. Similarly, information regarding iron supplement consumption was available for only three datasets. Two-factor interaction of each predictor variable with malaria infection was tested. Interactions with *P* > 0·1 were removed from the model. Collinearity was examined with the variable inflation factor (VIF), which determines the strength of the correlation between independent variables. A VIF > 5 indicated high collinearity. The coefficients from the linear model M were used to calculate the malaria-adjusted biomarker concentrations and to estimate the prevalence of malaria-adjusted deficiency with the equation:

log(malaria-adjusted biomarker) = inflammation-adjusted biomarker + *β̂*(malaria) + *β̂*_*i*_(interactions) + survey identifier

When adding an interaction in the model, the main effect of each variable included in the interaction is also included in the model. Model checking was based on residual and normal probability plots. In the models presented in these analyses, the residual plots showed no fitted pattern, and linear relationship between the predictors and the outcome variables was assumed. The residuals were spread equally along the ranges of predictors, suggesting no heteroscedasticity. The normality assumption was checked with the QQ plot of residuals. All analyses were performed using R Statistical Software (v4.1.2; R Core Team 2022)^(37)^. Individual survey analyses, accounting for the complex survey design, were performed with the use of the ‘survey’ package in R 4.2.1 software^(38)^. Collinearity was assessed with the package ‘car’^(39)^. The tables and graphs were made with the packages ‘kableExtra’, ‘interactions’ and ‘sjPlot’^(40–42)^. The protocol was reviewed and approved by the LSHTM observation research ethics committee (study reference 28219).

## Results

The pooled database included 7886 PSC. Among them, 1117 children did not have ferritin data and were excluded. Among the remaining children, 116 did not have results for a malaria test and were also excluded. These 116 children had significantly higher ferritin concentrations compared with children with a malaria test (geometric mean (μg/l) and 95 % CI 42 (39·5, 44·8) *v*. 32 (31·6, 33·2), *P* = 0·003). Additional missing information included the sex of children (*n* 22) and the type of residence (urban or rural) (*n* 10), but these children remained in the dataset. A total of 6653 children were in the final dataset (online Supplementary Table 1).

Table 2 displays participant demographics and prevalence of iron deficiency from each survey and for the entire sample set, and Table 3 displays prevalence of malaria in the entire sample set by sex, age group, rural and urban region, malaria endemicity, and malaria diagnostic method. The children’s mean age was 26·4 months. The unweighted pooled prevalence of malaria was 26·0 % (95 % CI 25·0, 27·1). The prevalence of malaria was higher in the older age group and in rural areas (Table 3). Iron deficiency, defined as inflammation-adjusted ferritin < 12 µg/l, ranged from 17·3 % in Zambia to 72·4 % in the Kenya 2007 dataset. The non-weighted pooled iron deficiency prevalence was 41·9 % (Table 2).

**Table 2.** Participant characteristics (weighted percentage or mean) of children aged 6–59 months from eight datasets from the BRINDA database in malaria endemic countries in Africa (*n* 6653)

Country	*n*	Male (%)	95 % CI	Age in months (mean)	95 % CI	Rural (%)	95 % CI	Iron deficiency (%)	95 % CI	Positive malaria test (%)	95 % CI
Cote d’Ivoire 2007 (6–59 months)	733	46·1	42·3, 49·9	31·7	30·4, 32·9	48·7	36·7, 60·9	39·2	35·0, 43·5	27·2	22·0, 33·0
Cameroon 2009 (12–59 months)	771	49·8	46·3, 53·3	30·7	29·8, 31·6	41·3	31·2, 52·2	34·6	30·0, 39·5	25·8	20·5, 32·0
Kenya 2007 (6–36 months)	888	47·4	44·4, 50·4	19·9	19·3, 20·6	100·0	100·0, 100·0	72·4	68·6, 75·9	19·7	16·1, 23·8
Kenya 2010 (6–35 months)	843	49·7	46·3, 53·1	21·4	20·8, 22·0	100·0	100·0, 100·0	53·1	49·4, 56·8	32·5	28·5, 36·7
Liberia 2011 (6–36 months)	1434	49·6	46·8, 52·4	19·9	19·5, 20·4	62·5	54·8, 69·6	51·0	47·0, 55·0	29·4	25·9, 33·1
Malawi 2016 (6–59 months)	1084	50·9	47·8, 54·0	32·5	30·8, 34·1	90·2	74·5, 96·6	21·2	16·7, 26·5	27·9	20·7, 36·4
Nigeria 2012 (6–59 months)	495	50·8	45·1, 56·5	30·7	29·1, 32·2	100·0	100·0, 100·0	18·4	14·0, 23·7	35·2	29·6, 41·2
Zambia 2009 (6–59 months)	405	42·2	36·4, 48·3	35·0	30·8, 39·2	77·2	58·9, 88·9	17·3	12·1, 24·1	18·5	13·2, 25·4
Total	6653	48·5	47·3, 49·7	26·4	26·1, 26·8	71·8	70·8, 72·9	41·9	40·7, 43·1	26·0	25·0, 27·1

Iron deficiency is defined by ferritin inferior to 12 μg/l. Ferritin concentrations were adjusted for inflammation with the BRINDA regression method^(18)^. Malaria testing was done with microscopy in Kenya, Cote d’Ivoire, Nigeria and Zambia, and rapid diagnostic tests in the other countries (Pf rapid-diagnostic test, Orchid Biomedical System in Liberia, Malaria Ag CELISA kit, Cellabs Pty, Ltd in Cameroon, BIOLINE Malaria Ag *P.f*/Pan in Malawi. These three kits detect the presence of histidine-rich protein 2). The results in the total line are not weighted as the total is not meant to be representative at any regional level.

**Table 3.** Prevalence of malaria by study characteristics (unweighted percentage) of children aged 6–59 months from eight datasets from the BRINDA database in malaria endemic countries in Africa (*n* 6653)

	Number of observations	Prevalence of malaria	95 % CI	*P*
Sex				
Male	3415	26·6	25·1, 28·1	0·4
Female	3216	25·6	24·1, 27·2	
Age group				
6–24 months	3020	21·6	20·2, 23·1	< 0·001
25–59 months	3633	29·8	28·3, 31·3	
Residence				
Rural	4773	30·1	28·9, 31·5	< 0·001
Urban	1870	15·7	14·1, 17·4	
Malaria endemicity profile				
Moderate endemicity	4486	26·3	25, 27·6	0·5
High endemicity	2167	25·6	23·8, 27·4	
Malaria diagnostic method				
RDT	3305	25·6	24·1, 27·1	0·4
Microscopy	3348	26·5	25·1, 28	
Total	6653	26·0	25·0, 27·1	

RDT, rapid diagnostic test; PfP, *Plasmodium falciparum* infection prevalence.

The results in the total line are not weighted as the total is not meant to be representative at any regional level. The malaria endemicity profile was defined by the *P. falciparum* infection prevalence among children aged 2–10 years, as described in the Malaria Atlas Project. Moderate endemicity was defined by a PfP ≥ 10 % and < 35 %, and high endemicity was defined by a PfP ≥ 35%. Malaria testing was done with microscopy in Kenya, Cote d’Ivoire, Nigeria and Zambia, and rapid diagnostic tests in the other countries (Pf rapid-diagnostic test, Orchid Biomedical System in Liberia, Malaria Ag CELISA kit, Cellabs Pty, Ltd in Cameroon, BIOLINE Malaria Ag *P.f*/Pan in Malawi. These three kits detect the presence of histidine-rich protein 2).

### Crude analysis

In the crude analysis, inflammation-adjusted ferritin concentration was significantly higher in children with malaria infection than in those uninfected: 18·7 (95 % CI 17·9, 19·4) µg/l *v*. 12·3 (95 % CI 11·9, 12·6) µg/l, *P* < 0·001, analysis done on the log scale.

Table 4 presents the inflammation-adjusted geometric mean ferritin concentrations by country, age group, residence and malaria endemicity profile. The older age group had a higher inflammation-adjusted ferritin than the younger age group (17·5 *v*. 10·2 µg/l). Ferritin was higher in the rural areas and in the settings of moderate malaria endemicity profile compared with the urban areas and the settings of high endemicity profile. There was no significant difference by malaria diagnosis method.

**Table 4.** Inflammation-adjusted ferritin concentration (µg/l) per participant and study characteristics among children aged 6–59 months from eight datasets from the BRINDA database in malaria endemic countries in Africa (*n* 6653)

	Number of observations	Geometric mean	95 % CI	*P*
Country				
Cote d’Ivoire	733	15·0	14·0, 16·0	< 0·001
Cameroon	771	14·5	13·7, 15·3	
Kenya 2007	888	6·6	6·2, 7·0	
Kenya 2010	843	10·3	9·7, 11·0	
Liberia	1434	10·8	10·3, 11·3	
Malawi	1084	23·3	22·3, 24·4	
Nigeria	495	21·9	20·5, 23·3	
Zambia	405	29·0	26·5, 31·8	
Sex				
Male	3415	13·2	12·8, 13·7	0·2
Female	3216	14·2	13·7, 14·6	
Age group				
6–24 months	3020	10·2	9·8, 10·5	< 0·001
25–59 months	3633	17·5	17·0, 18·0	
Residence				
Rural	4773	13·9	13·6, 14·3	< 0·001
Urban	1870	12·9	12·4, 13·4	
Malaria endemicity profile				
Moderate endemicity	4486	14·5	14·1, 15·0	< 0·001
High endemicity	2167	12·1		
Malaria diagnosis method				
RDT	3305	14·9	14·5, 15·4	0·5
Microscopy	3348	12·5	12·1, 13·0	

RDT, rapid diagnostic test. PfP, *Plasmodium falciparum* infection prevalence.

Inflammation adjustment was done with the BRINDA method^(18)^. The malaria endemicity profile was defined by the *P. falciparum* infection prevalence among children aged 2–10 years, as described in the Malaria Atlas Project. Moderate endemicity was defined by a PfP ≥ 10 % and < 35 %, and high endemicity was defined by a PfP ≥ 35%. Malaria testing was done with microscopy in Kenya, Cote d’Ivoire, Nigeria and Zambia, and rapid diagnostic tests in the other countries (Pf rapid-diagnostic test, Orchid Biomedical System in Liberia, Malaria Ag CELISA kit, Cellabs Pty, Ltd in Cameroon, BIOLINE Malaria Ag *P.f*/Pan in Malawi. These three kits detect the presence of histidine-rich protein 2).

### Association between malaria and ferritin

Table 5 presents the results of the regression analysis to estimate the association between malaria and inflammation-adjusted ferritin. The relative difference in inflammation-adjusted ferritin concentration between children with malaria infection and those uninfected was 44 % (95 % CI 39, 52) after adjusting for age and survey identifier. The measure of the main association between ferritin and malaria is derived from model A, as it represents the main effect of malaria on ferritin, with the survey considered as a fixed effect (online Supplementary Table 2, model A). The difference in ferritin concentration between children with malaria infection and those without was significant in both unadjusted analyses and those adjusted for each of the potential confounders (online Supplementary Table 2). Age was the only potential cofounder that modified the effect estimate for the main exposure; therefore, age was kept in each model (+ 40 %, 95 % CI 35·0, 46·0, *P* < 0·001). Adjusting for multiple cofounders did not modify the value of the main estimate for malaria on ferritin concentration derived from model A (online Supplementary Table 3).

**Table 5.** Difference in inflammation-adjusted ferritin concentration (log ferritin, µmol/l) between children with malaria infection and those without infection in the multivariable linear regression models among children aged 6–59 months from eight datasets in malaria endemic countries in Africa (*n* 6653)

	Difference in log ferritin	95 % CI	Difference in ferritin	95 % CI	*P*
**Model A** Multivariable linear regression analysis to estimate the association between malaria and inflammation-adjusted ferritin, adjusted for survey ID and age group					
Malaria	0·37	0·32, 0·41	44 %	39 %, 52 %	< 0·001
Age group (> 2 years *v*. < 2 years)	0·34	0·30, 0·38	40 %	35 %, 46 %	< 0·001
R^2^:0·26 *P* < 0·001					
**Model M** Multivariable linear regression analysis to estimate the association between malaria and inflammation-adjusted ferritin – including the main effect of age group and malaria endemicity profile and the interactions between age group and malaria and between malaria endemicity profile and malaria					
Malaria	0·52	0·44, 0·61	69 %	55 %, 84 %	< 0·001
Age group (> 2 years *v*. < 2 years)	0·54	0·49, 0·6	72 %	63 %, 82 %	< 0·001
Malaria endemicity profile (high *v*. moderate)	–0·07	–0·13, −0·2	–7 %	–12 %, −2 %	0·008
Interaction Malaria*age group (> 2 years *v*. < 2 years)	–0·16	–0·26, −0·06	–15 %	–23 %, −6 %	*P* for the interaction 0·002
Interaction Malaria*endemicity profile (high *v*. moderate)	–0·19	–0·29, −0·08	–17 %	–25 %, −8 %	*P* for the interaction < 0·001
R^2^:0·12 *P* < 0·001					

Inflammation adjustment was done with the BRINDA method^(18)^. Including endemicity profile in the model resulted in a high level of collinearity with study identifier; therefore, model M is run without the study identifier.

### Prevalence of iron deficiency after adjustment for inflammation and malaria

Table 6 presents estimates of iron deficiency using different adjustment methods (Table 6). Adjusting for malaria with any method, in addition to inflammation only, would increase the prevalence of iron deficiency in all countries, by less than 10 percentage points (Table 6).

**Table 6.** Prevalence of iron deficiency per country per adjustment method among children aged 6–59 months from eight datasets from the BRINDA database in malaria endemic countries in Africa (*n* 6653)

Country	Iron deficiency (%) and 95 % CI without any adjustment	95 % CI	Iron deficiency (%) and 95 % CI using BRINDA adjustment only	95 % CI	Iron deficiency (%) and 95 % CI using BRINDA and malaria without interactions	95 % CI	Iron deficiency (%) and 95 % CI using BRINDA and malaria regression adjustment	95 % CI
Cote d’Ivoire	11·7	9·0, 15·1	39·2	35·0, 43·5	44·3	40·2, 48·4	41·4	37·2, 45·8
Cameroon	14·9	12·0, 18·4	34·6	30·0, 39·5	39·6	35·1, 44·2	39·8	35·4, 44·4
Kenya 2007	38·9	34·8, 43·1	72·4	68·6, 75·9	75·8	72·3, 78·9	76·4	72·8, 79·6
Kenya 2010	19·2	16·1, 22·8	53·1	49·4, 56·8	61·1	57·5, 64·5	61·4	57·7, 65·1
Liberia	20·4	17·9, 23·2	51·0	47·0, 55·0	57·5	53·7, 61·1	54·9	51·0, 58·8
Malawi	10·8	7·8, 14·8	21·2	16·7, 26·5	24·0	19·8, 28·8	23·9	19·7, 28·6
Nigeria	5·3	3·5, 7·9	18·4	14·0, 23·7	23·6	18·6, 29·5	24·0	19·0, 30·0
Zambia	5·4	2·8, 10·3	17·3	12·1, 24·1	18·3	13·1, 24·8	18·5	13·4, 25·0

Iron deficiency is defined by serum ferritin concentration below 12 µg/l. Inflammation adjustment was done with the BRINDA regression method^(18)^. Survey weights are applied to account for the survey design. The adjustment using BRINDA and malaria without interactions is based on model A. The malaria regression adjustment is using a linear model with the interactions between malaria and age group, and between malaria and endemicity profile (model M).

### Effect modifiers in the association between malaria and ferritin

**Fig. 1.**
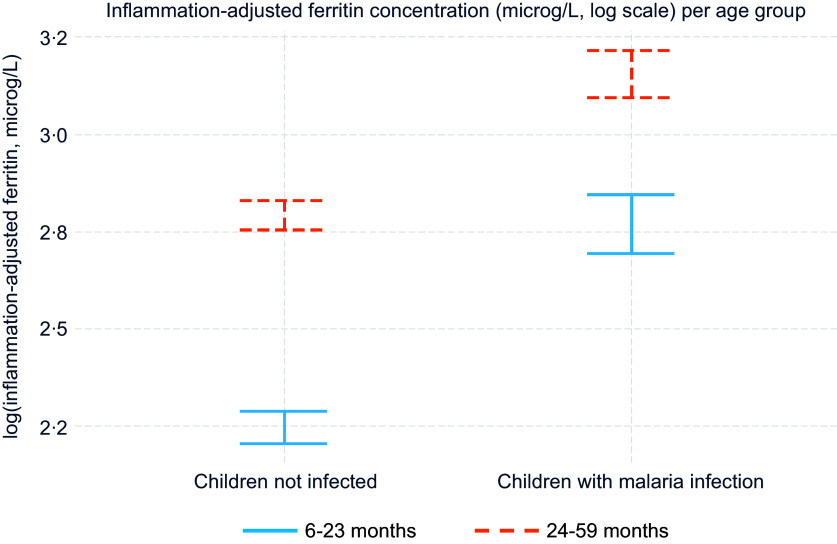
Mean inflammation-adjusted ferritin concentration (log scale, µg/l) by age group among children with malaria infection (*n* 1733) and children not infected (*n* 4920) among children aged 6–59 months from eight datasets from the BRINDA database in malaria endemic countries in Africa. Inflammation adjustment was done with the BRINDA method^(18)^. The model is also adjusted for the interaction between malaria and endemicity level (model M). Effect estimate of age on inflammation-adjusted ferritin concentration in children with malaria infection (> 2 years *v*. < 2 years): −15 % (–23 %, −6 %). *P* for interaction malaria * age: 0·002.

**Fig. 2.**
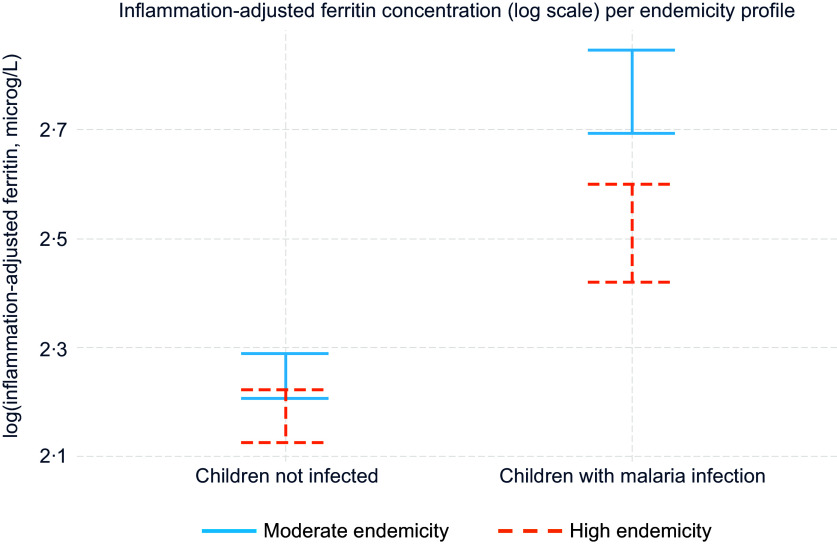
Mean inflammation-adjusted ferritin concentration (log scale, µg/l) per endemicity profile in children with malaria infection (*n* 1733) and children not infected (*n* 4920) – among children aged 6–59 months from eight datasets from the BRINDA database in malaria endemic countries in Africa. Inflammation adjustment was done with the BRINDA method^(18)^. The model is also adjusted for the interaction between malaria and age (model M). Effect estimate of malaria endemicity profile on inflammation-adjusted ferritin concentration in children with malaria infection (high endemicity *v*. moderate endemicity): −17 % (–25 %, −8 %). *P* for interaction malaria * endemicity profile: < 0·001.

There were negative and significant interactions between malaria infection, defined as a binary variable (infected or uninfected), and age and between malaria and endemicity profile on inflammation-adjusted ferritin concentration (Table 5, model M). Figures 1 and 2 present inflammation-adjusted ferritin concentration in children with malaria infection and children without malaria infection, by endemicity profile and by age (Figs. 1 and 2). Among children with malaria infection compared with those uninfected, ferritin was less elevated in older children and those in higher endemicity profile compared with younger children and those in moderate endemicity profile (Figs. 1 and 2). Adding other potential cofounders in the model did not modify the effect estimate for malaria on ferritin (online Supplementary Table 3). None of the other tested interactions were significant (online Supplementary Table 2).

### Stratified analysis by endemicity profile

**Fig. 3. f3:**
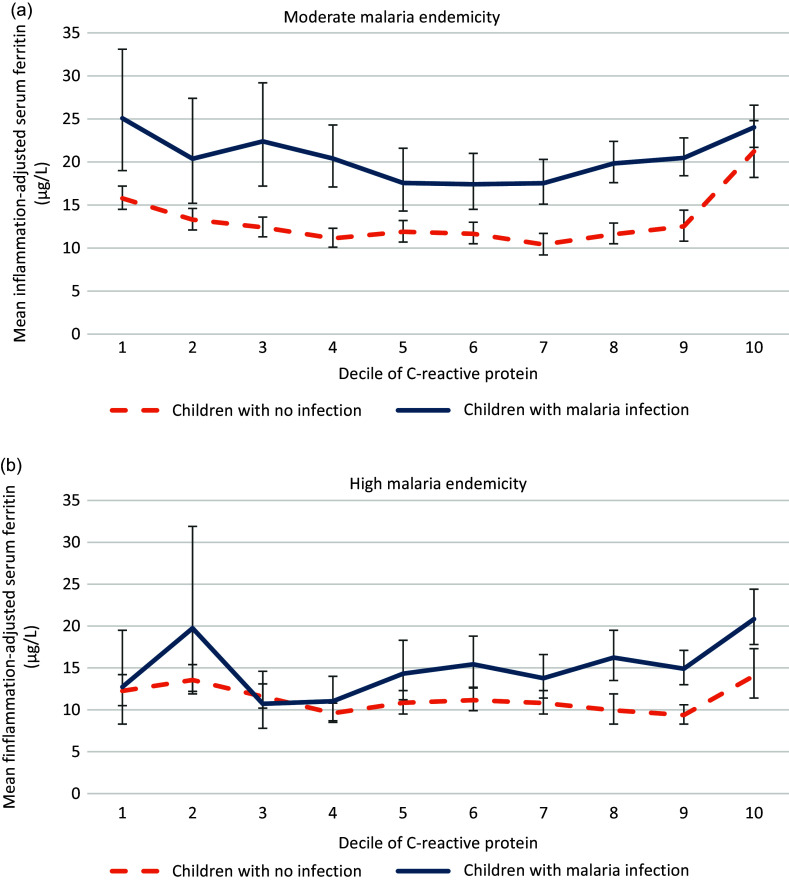
Inflammation-adjusted ferritin concentration (µg/l, geometric mean and 95 % CI) per CRP decile in children with malaria infection and children not infected in (a) moderate (*n* 4486) and (b) high (*n* 2167) endemicity profile among children aged 6–59 months from eight datasets from the BRINDA database in malaria endemic countries in Africa. Inflammation adjustment was done with the BRINDA method^(18)^. The CRP deciles were derived from the entire dataset. The maximum value of each decile is: 1st decile: 0·2 mg/l, 2nd decile: 0·4 mg/l, 3rd decile: 0·7 mg/l, 4th decile: 1·2 mg/l, 5th decile: 2 mg/l, 6th decile: 3·4 mg/l, 7th decile: 5·9 mg/l, 8th decile: 13 mg/l, 9th decile: 24 mg/l, 10th decile: 864 mg/l. The difference between inflammation-adjusted ferritin in children with malaria infection and children without malaria infection is positive and statistically significant (*P* < 0·05) in (a) at every decile except the 10th decile and in (b) at deciles 6, 8, 9 and 10. Statistical significance was assessed with a linear model that included the main effect of malaria and the interaction between malaria and age. CRP, C-reactive protein.

Figure 3 presents mean inflammation-adjusted ferritin concentration by decile of CRP values, by endemicity profile, in children with malaria infection and children without malaria infection[Fig f3]. In moderate endemicity, inflammation-adjusted ferritin concentrations were consistently higher in children with malaria infection, at every decile of CRP (Fig. 3). In high endemicity, inflammation-adjusted ferritin values between malaria groups were very similar at low level of CRP. From the 5th–6th decile of CRP (CRP values between 1·2 and 3·4 mg/l), inflammation-adjusted ferritin concentrations started to differ between children with malaria infection and children who were not infected, and the difference was statistically significative at deciles 6, 8, 9 and 10. The same figure with non-adjusted ferritin concentrations showed a similar pattern, with visible increase in ferritin in children with malaria infection and children who were not infected from the 5th decile of CRP (online Supplementary Fig. 1).

### Sub-group analysis on ferritin and malaria parasitaemia

Three datasets had data on malaria parasitaemia (number of parasites per µl of blood): Cote d’Ivoire, Kenya 2007 and Kenya 2010. The overall mean parasite density was 204 parasites/µl of blood, and 63 % of children had a low level of parasitaemia (< 1000 parasites/µl of blood). Table 7 presents inflammation-adjusted ferritin concentration where malaria parasitemia was available[Table tbl7].

**Table 7. tbl7:** Malaria parasitaemia and inflammation-adjusted ferritin concentration by country among children aged 6–59 months from three datasets from the BRINDA database in malaria endemic countries in Africa (*n* 1809)

	Kenya (combined 2007 and 2010 datasets)				Cote d’Ivoire			
Malaria parasitaemia	*n*	Inflammation-adjusted serum ferritin, mean (µg/l)	95 % CI	*P* (compared to children without infection)	*n*	Inflammation-adjusted serum ferritin, mean (µg/l)	95 % CI	*P* (compared to children without infection)
Uninfected children	1282	7·1	6·8, 7·5		527	14·2	13·1, 15·4	
Low (< 1000 parasites/µl of blood)	151	12·3	10·7, 14·2	< 0·001	126	16·6	14·3, 19·2	0·07
Intermediate (1000–10 000 parasites/µl of blood)	156	11·3	9·9, 12·7	< 0·001	65	16·5	132, 20·7	0·07
High (> 10 000 parasites/µl of blood)	142	13·2	11·7, 14·9	< 0·001	15	27·8	17·5, 44·2	0·01

In Kenyan children with low parasitaemia, inflammation-adjusted ferritin concentration was elevated by 73 % relative to uninfected children (*P* < 0·001) whereas in Cote d’Ivoire, the increase was not as large (21 %, *P* = 0·07). In Cote d’Ivoire, only high parasitaemia was associated with a significant increase in inflammation-adjusted concentration (100 % relative increase, *P* = 0·01) (Table 7).

In Kenya, where the mean age of children was about 20 months, the difference in ferritin between children without infection and children with malaria was higher in young children compared with older children (P_for interaction_: 0·02). This was not the case in Cote d’Ivoire, where the mean age of children was 32 months.

## Discussion

Our analysis showed that children with malaria infection had higher inflammation-adjusted ferritin concentrations than children without infection. This association held after allowing for the confounding effects of age, sex and rural–urban residency. These results are consistent with studies in Zambia^(17)^, Burkina Faso^(16)^ and a pooled analysis of children in Gambia, Uganda, Burkina Faso and Kenya^(43)^. This indicates that estimates of iron deficiency based on inflammation-adjusted ferritin only should be interpreted with caution in malaria endemic areas. Based on our model that adjusted for age and survey identifier, a malaria infection in PSC is associated with a 44 % (95 % CI 39, 52) increase in inflammation-adjusted ferritin concentration. Adjusting for malaria increased the prevalence of iron deficiency in all countries, and the increase was significant in one Kenya dataset.

At least two mechanisms may explain a greater inflammation-adjusted ferritin concentration in children with malaria infection. First, during a malaria infection, ferritin is increased with CRP and AGP but stays elevated for longer than CRP and AGP, possibly due to a longer half-life of ferritin^(4)^. Second, ferritin can be elevated independently of CRP and AGP during a malaria infection. The elevation could be either through an inflammatory pathway that is not captured by AGP and CRP, or via a non-inflammatory pathway. These two mechanisms would involve a redistribution of body iron.

The difference in ferritin concentration between children with malaria infection and children without was greater in the younger children (< 2 years) compared with the older children (2–5 years) and greater in moderate compared with high endemicity settings. This difference by age group was confirmed in the sub-group analysis per malaria parasitaemia in the two Kenya datasets, where we saw that ferritin increased more in younger children compared with older children when malaria parasitaemia increased. Stratified analyses showed different patterns in moderate and high endemicity profiles. In moderate endemicity, ferritin concentrations were higher in children with malaria infection at every decile of CRP, suggesting that even infections causing a low level of inflammation – possibly asymptomatic infections – were associated with a ferritin concentration increase. The ferritin concentration difference between groups with malaria infection and non-infection was fairly constant at every decile of CRP in areas of moderate endemicity. In areas with high endemicity, there was no difference in ferritin concentration between children with malaria infection and children without infection at low levels of CRP. A marked difference could be seen from the 5th–6th centile of CRP that corresponds in our dataset to CRP values between 1·2 and 3·4 mg/l. The different pattern between high and moderate endemicity could be interpreted as an adaptation and acquired immunity to malaria in areas of high endemicity^(44)^. In high endemicity, it appears that infections with lower levels of inflammation, probably asymptomatic infections, were not associated with increased ferritin once ferritin was adjusted for CRP and AGP. A similar trend was observed in a study of PSC on Pemba Island, Zanzibar, where *P. falciparum* was holoendemic^(36)^. Serum ferritin was higher with higher malaria parasitaemia in younger children, but there was no association between serum ferritin and malaria in older children. The authors speculated that age-dependent immune mechanisms might have protected older children. A study involving school-age children also conducted in Zanzibar^(45)^ showed no relation between ferritin and malaria at low infection levels, characterised by low parasitaemia (< 1000 parasites/µl blood). Above this cutoff, ferritin increased slightly with parasitaemia. This seems to mirror our findings from high endemicity settings, wherein malaria was associated with increased ferritin only at higher CRP levels.

The sub-group analysis per parasitaemia showed different patterns in the two countries which had available data. In Kenya, a country with moderate endemicity, even a low parasitaemia was associated with an elevation in inflammation-adjusted ferritin, which corresponds to what we observed at low levels of CRP in moderate malaria endemicity. In Cote d’Ivoire, a country with high malaria endemicity, only a high parasitaemia was associated with a clinically relevant increase in inflammation-adjusted ferritin. This corresponds to what was observed in high endemicity settings, where inflammation-adjusted ferritin was increased only at high CRP levels. This is also consistent with the findings from the above-mentioned study in Zanzibar^(45)^. Although there are other factors that should be considered when comparing these two countries, such as younger mean age of children in Kenya compared with Cote d’Ivoire, this analysis of parasitaemia supports our findings related to the importance of endemicity and acquired immunity. This analysis also indicates that measuring parasitaemia may help elucidate the effect of malaria on iron status, or the severity of the malaria infection – as the relationship between parasitaemia and inflammation-adjusted ferritin does not seem linear and comparable in different settings. It would be worth exploring whether other inflammatory markers could be more related to parasitemia and could be used to adjust ferritin for inflammation, in addition to AGP and CRP.

These results bring some nuance to the previous ferritin analysis of BRINDA datasets^(5)^. In 2017, five PSC datasets were analysed, and malaria infection was independently associated with ferritin after adjusting for CRP and AGP. However, adjusting for malaria alone, or adjusting for malaria in addition to CRP and AGP, did not significantly change the prevalence estimates of iron deficiency. While this conclusion was valid in the 2017 dataset, it is worth noting that the estimates of iron deficiency prevalence could be affected differently in other settings. Our analysis indicated that age and endemicity modify the association between malaria and ferritin; therefore adjusting for malaria is likely to change the prevalence of iron deficiency in certain contexts, particularly in young children in moderate malaria endemicity settings. Our analysis indicates that adjusting for malaria with a regression analysis would increase the estimated prevalence of iron deficiency in all countries, although the effect was small. Even moderate changes in the estimates of iron deficiency prevalence can have important consequences in terms of public spending, whereby decisions are made on the basis of the severity of the public health problems or on cost–benefit analyses (often relying on thresholds). Moreover, it is important to understand the factors that impact the concentration of serum ferritin, as it is the most used indicator of iron status and is often analysed as a continuous variable in longitudinal analyses. Other factors could be considered, such as parasite species and patterns of transmission. Although the use of different diagnostic methods for malaria can detect different stages of infections, the diagnostic method did not appear to modify the association between ferritin and malaria in our database. As RDT can be positive for several weeks after parasite clearance due to persistent antigenemia^(14)^, we would expect to see higher ferritin concentrations in malaria cases detected by RDT compared with microscopy. However, it is usually believed that microscopy can detect earlier cases of malaria^(14)^, which would result in higher ferritin concentrations as well, and therefore the two mechanisms might counteract each other. To truly assess the potential cofounding effect of the malaria diagnostic test on the association between ferritin and malaria, we would need to analyse ferritin concentrations in surveys where the two diagnostic methods are used concomitantly. This analysis also highlights the lack of sensitivity of ferritin status as an indicator of iron deficiency and questions whether other inflammatory conditions (obesity and COVID) could result in an increase in inflammatory-adjusted ferritin.

### Strengths and limitations

A major strength of this study is the availability of individual-level data on malaria infection and iron status from seven countries, resulting in a large pooled sample size. Regarding limitations, since the data are cross-sectional, we cannot ascertain the order of events, for instance whether children had higher ferritin at the time they were infected by malaria. However, prospective studies on malarial infection and indicators of iron status^(15,46,47)^ suggested a causal effect of malaria on ferritin levels and showed that ferritin concentrations tend to return to pre-infection level about 3 weeks after the infection. Furthermore, the moderate and high endemicity categorisation profile was done at the country level, as localisation data were not available for national surveys. Subnational data on endemicity levels could add precision to the analysis. The datasets were limited to those with cofounders or effect modifiers that were available for all countries; thus, fever in the last 24 h, maternal education and iron supplement consumption were excluded from the final model. Also, about 15 % of the sample was not included in the analysis because of missing ferritin data, which could have introduced bias into representativity.

### Conclusion

In our database of 6653 pre-school-aged children from seven countries, children with malaria infection had higher inflammation-adjusted ferritin concentrations than children not infected. The results suggest a specific serum ferritin response to malaria infection, which seems to be lower in older children and in areas of high malaria endemicity. Inflammation-adjusted ferritin using only CRP and AGP might be a valid indicator of iron deficiency in population groups with high immunity to malaria; but in groups with lower levels of immunity, inflammation-adjusted ferritin using only CRP and AGP might not address the independent effect of malaria on ferritin concentration. A similar analysis in other population groups with higher immunity (school-age children and non-pregnant adults) could help check these assumptions. Analysing the association between malaria infections and other indicators, such as soluble transferrin receptors and Hb, could help clarify iron status in settings with different malaria endemicity.

## Supporting information

Sandalinas et al. supplementary material 1 Sandalinas et al. supplementary material

Sandalinas et al. supplementary material 2 Sandalinas et al. supplementary material

Sandalinas et al. supplementary material 3 Sandalinas et al. supplementary material

Sandalinas et al. supplementary material 4 Sandalinas et al. supplementary material

## Supplementary material

For supplementary material/s referred to in this article, please visit https://doi.org/10.1017/S0007114524002319
