# Supplementary material for: Current or recent malaria infection is associated with elevated inflammation-adjusted ferritin concentrations in pre-school children: a secondary analysis of the BRINDA database
Source: Br J Nutr. 2024 Oct 25;132(8):1093–103. doi: 10.1017/S0007114524002319 (PMC11600282; doi:10.1017/S0007114524002319)
Supplement: Sandalinas et al. supplementary material 1 — Sandalinas et al. supplementary material [file S0007114524002319sup001.docx]

**Supplementary Fig. 1.** Ferritin concentration (µg/L, geometric mean and 95 % CI) per CRP decile in children with malaria infection and children not infected, in (a) moderate (n 4486) and (b) high endemicity (n 2167) profile, among children aged 6–59 months from eight datasets from the BRINDA database in malaria endemic countries in Africa.

CI, confidence interval, CRP, C-reactive protein.
