# Supplementary material for: Current or recent malaria infection is associated with elevated inflammation-adjusted ferritin concentrations in pre-school children: a secondary analysis of the BRINDA database
Source: Br J Nutr. 2024 Oct 25;132(8):1093–103. doi: 10.1017/S0007114524002319 (PMC11600282; doi:10.1017/S0007114524002319)
Supplement: Sandalinas et al. supplementary material 2 — Sandalinas et al. supplementary material [file S0007114524002319sup002.docx]

**Supplementary Table 2.** Difference in ferritin concentration (log ferritin, µmol/L) between children with malaria infection and those without infection, adding each cofounder and each two-factor interaction in turn, among children aged 6–59 months from eight datasets from the BRINDA database in malaria endemic countries in Africa (*n* 6653)

| **Model** | **Potential cofounder** | **Difference in log ferritin concentration between children with malaria infection and children not infected (95 % CI)** | **Difference in ferritin concentration between children with malaria infection and children not infected (95 % CI)** | ***P* value of the model**  (and *P* value of the interaction for model G-I) |
| --- | --- | --- | --- | --- |
| Base | Study ID  (*n* 8292) | 0·40 (0·35, 0·44) | 49 % (42 %, 55 %) | < 0·001 |
| A | Study ID, Age (*n* 8292) | 0·37 (0·32, 0·41) | 44 % (38 %, 51 %) | < 0·001 |
| B | Study ID, Sex (*n* 8270) | 0·40 (0·36, 0·45) | 49 % (43 %, 57 %) | < 0·001 |
| C | Study ID, Residence  (*n* 8282) | 0·39 (0·35, 0·44) | 48 % (42 %, 55 %) | < 0·001 |
| D | Endemicity profile  (*n* 8282) | 0·42 (0·37, 0·47) | 50 % (45 %, 60 %) | < 0·001 |
| E | Diagnostic method  (*n* 8282) | 0·34 (0·29, 0·39) | 40 % (34 %, 48 %) | < 0·001 |
| F | Study ID ,  Interaction malaria*age  (*n* 8292) | 06–24 months: 0·43 (0·35, 0·51)  25–59 months: 0·34 (0·28, 0·39) | 54 % (42 %, 67 %)  41 % (32 %, 48 %) | < 0·001  *P* for the interaction: 0·005 |
| G | Study ID,  Interaction malaria*sex  (*n* 8270) | girls: 0·41 (0·34, 0·47)  boys: 0·39 (0·33, 0·46) | 51 % (40 %, 60 %)  48 % (39 %, 58 %) | < 0·001  *P* for the interaction: 0·9 |
| H | Interaction  malaria*endemicity profile  (*n* 6653) | Moderate endemicity: 0·46 (0·40, 0·53)  High endemicity: 0·33 (0·24, 0·41) | 58 % (49 %,70 %)  39 % (27 %, 51 %) | < 0·001  *P* for the interaction: 0·015 |
| I | Interaction  malaria*diagnostic method  (*n* 8282) | RDT: 0·49 (0·43, 0·56)  Microscopy: 0·36 (0·28, 0·44) | 63 % (54 %,75 %)  43 % (32 %, 55 %) | < 0·001  *P* for the interaction: 0·5 |

CI, confidence interval; RDT, rapid diagnostic test. The variables’ endemicity profile and malaria diagnostic method had a high level of collinearity with the survey identifier. Therefore, models D, E, H and I were run without the fixed adjustment for survey identifier.
