# Supplementary material for: Current or recent malaria infection is associated with elevated inflammation-adjusted ferritin concentrations in pre-school children: a secondary analysis of the BRINDA database
Source: Br J Nutr. 2024 Oct 25;132(8):1093–103. doi: 10.1017/S0007114524002319 (PMC11600282; doi:10.1017/S0007114524002319)
Supplement: Sandalinas et al. supplementary material 3 — Sandalinas et al. supplementary material [file S0007114524002319sup003.docx]

**Supplementary Table 3.** Difference in ferritin concentration (log ferritin, µmol/L) between children with malaria infection and children not infected, adding multiple cofounders and two-factor interactions, among children aged 6–59 months from eight datasets from the BRINDA database in malaria endemic countries in Africa (*n* 6653)

| **Model** | **Adjusted for** | ***N*** | **Difference in log ferritin (95 % CI)** | **Difference in ferritin (95 % CI)** | ***P* for the model** | ***P* for the interaction** |
| --- | --- | --- | --- | --- | --- | --- |
| J | Study ID, age, sex, residence | 8270 | 0·37 (0·32, 0·41) | 45 % (38 %, 51 %) | < 0·001 |  |
| K | Study ID, sex, residence  Interaction malaria* age | 8270 | 6–24 months: 0·44  (0·37, 0·52)  25–59 months:  0·32 (0·26, 0·37) | 56 % (45 %, 68 %)  38 % (30 %, 45 %) | < 0·001 | 0·004 |
| L | Sex, residence  Interaction malaria*age  Interaction malaria*endemicity profile | 6621 | 6–24 months, moderate endemicity:  0·61 (0·51, 0·71)  6–24 months, high endemicity:  0·29 (0·16, 0·41)  25–59 months, moderate endemicity  0·32 (0·25, 0·40)  25–59 months, high endemicity  0·16 (0·04, 0·27) | 84 % (67 %, 103 %)  34 % (17 %, 51 %)  38 % (28 %, 49 %)  17 % (4 %, 31 %) | < 0·001 | Malaria*age:  *P* = 0·002  Malaria*endemicity profile:  *P* = 0·002 |
| M | Interaction malaria*age  Interaction malaria*end profile | 6653 | 6–24 months, moderate endemicity:  0·57 (0·46, 0·67)  6–24 months, high endemicity:  0·27 (0·15, 0·40)  25–59 months, moderate endemicity  0·32 (0·24, 0·39)  25–59 months, high endemicity  0·30 (0·19, 0·41) | 77 % (58 %, 95 %)  31 % (16 %, 49 %)  38 % (27 %, 48 %)  35 % (21 %, 51 %) | < 0·001 | Malaria*age:  *P* = 0·002  Malaria*endemicity profile:  *P* < 0·001 |

CI, confidence interval. * Including diagnostic method and endemicity profile in the model resulted in a high level of collinearity with study identifier, therefore models L and M were run without the study ID.
