# Supplementary material for: Current or recent malaria infection is associated with elevated inflammation-adjusted ferritin concentrations in pre-school children: a secondary analysis of the BRINDA database
Source: Br J Nutr. 2024 Oct 25;132(8):1093–103. doi: 10.1017/S0007114524002319 (PMC11600282; doi:10.1017/S0007114524002319)
Supplement: Sandalinas et al. supplementary material 4 — Sandalinas et al. supplementary material [file S0007114524002319sup004.docx]

BRINDA database:

30 datasets from 26 countries

Missing malaria result: 116

Missing ferritin values: 1117

Pre-school children with ferritin value: 6769

Pre-school children with ferritin value and malaria infection result: 6653 observations

BRINDA database with the result of a malaria test:

10 datasets from 9 countries

Dataset with retrospective measure of malaria antibodies: 1 (Burkina Faso 2010)

Dataset with no cases of malaria: 1 (Nepal 2016)

BRINDA database with a diagnosis of recent or current malaria infection in pre-school children:

8 datasets from 7 countries, 7886 observations

Supplementary Table 1: Flow diagram of the included datasets and sample size.
